# Optimized protocol for translatome analysis of mouse brain endothelial cells

**DOI:** 10.1371/journal.pone.0275036

**Published:** 2022-09-28

**Authors:** Namsuk Kim, Mi-Hee Jun, Jin-Young Jeong, Won-Jong Oh

**Affiliations:** 1 Neurovascular Unit Research Group, Korea Brain Research Institute, Daegu, South Korea; 2 Department of Brain and Cognitive Sciences, Daegu Gyeongbuk Institute of Science and Technology, Daegu, Republic of Korea; Eötvös Loránd Research Network Biological Research Centre, HUNGARY

## Abstract

Brain endothelial cells (BECs) are important conduits that deliver oxygen and nutrients, protect parenchyma cells from toxins, and drain wastes to maintain brain homeostasis. Impairment of BECs has been implicated in diverse neurodegenerative diseases, including Alzheimer’s disease and Parkinson’s disease. Therefore, molecular analysis of BECs is important for understanding the molecular pathogenesis of these neurological diseases. Even though many transcriptome analyses for BECs have been developed, mRNA levels do not necessarily correlate with the levels of actively translated proteins. Translatome analysis using RiboTag mice, in which Rpl22, a ribosomal component, is tagged by the hemagglutinin epitope under Cre recombinase activation, could serve as an excellent tool that overcomes these caveats. However, implementation of this technique is limited by high noise-to-signal ratios as well as the low yield of mRNAs from BECs, which limits bulk gene expression analysis. In this study, we established a protocol to isolate highly pure mRNAs from BECs in the cortex of eight- to twelve-week-old male *Tie2-Cre; Rpl22*^*HA/HA*^ mice by using a cell strainer to trap blood vessels prior to immunoprecipitation. According to the results of RT–PCR, the specificity of the mRNA pools isolated by our protocol was much higher than that of the pools isolated by the standard protocol. We were also able to generate a high-quality cDNA library for RNA-seq with the small amount of mRNA isolated with our protocol. Thus, this optimized method will be useful for future studies of BECs at the molecular level.

## Introduction

Blood vessels are highly vascularized tissues that are essential for proper brain function. Blood vessels in the brain supply oxygen and energy and protect parenchyma cells from potential toxins [1, 2]. The brain endothelial cells (BECs) that line blood vessel walls constitute the blood–brain barrier (BBB), which stringently regulates the movement of molecules, ions, and cells between the blood and the parenchyma cells to maintain brain homeostasis [3]. BBB disruption, specifically an increase in BBB permeability and a reduction in cerebral blood flow, has been implicated in diverse neurological disorders, including Alzheimer’s disease, Parkinson’s disease, and amyotrophic lateral sclerosis [1, 4, 5]. To understand the molecular mechanisms underlying these diseases, transcriptome databases of brain endothelial cells have been developed [6–8].

Cellular diversity and structural complexity create barriers to understanding the function of BECs at the molecular level. Although the transcriptome analyses used in previous studies have yielded clear BEC expression profiles, their importance is limited by the fact that mRNA levels do not necessarily correlate with the levels of actively translated proteins [6, 7, 9]. To overcome these problems, a new translatome analysis, termed the RiboTag method, has been developed. This method can provide profiles of the translated mRNAs of genetically defined cell populations. RiboTag mice have an allele of the ribosomal protein *Rpl22* with a floxed wild-type exon 4 and consecutive exon 4 that contains the hemagglutinin (HA) epitope inserted before the stop codon [10]. When a RiboTag mouse is crossed with a specific Cre recombinase-expressing mouse, Cre recombinase replaces wild-type RPL22 with the HA-tagged ribosomal protein RPL22^HA^. Subsequent immunoprecipitation of polysomes with an antibody against the HA tag yields ribosome-associated mRNA pools from specific cell types [10]. However, there are limitations when applying the RiboTag method to BECs, such as the nonspecific binding of non-BEC mRNAs to the magnetic beads and the low yield of mRNA for bulk gene expression analysis in BECs.

Here, we developed an optimized protocol for BEC-specific translatome analysis by modifying the original RiboTag method. We added a blood vessel isolation process by using a cell strainer before immunoprecipitation. With this simple modification, we were able to increase the purity of BEC-specific mRNA pools compared to that of pools isolated with the original protocol. The BEC specificity of the isolated mRNA pools was validated by RT–PCR. Moreover, we adapted the protocol to generate a cDNA library for next-generation sequencing (NGS), enabling bulk translatome analysis of BECs. Altogether, we established an optimized RiboTag method for RNA-seq of BECs, which will be useful for researchers studying BECs at the molecular level and could be easily applied to translatome analysis of other types of brain cells.

## Materials and methods

The detailed RiboTag protocol described in this article is published in protocols.io, dx.doi.org/10.17504/protocols.io.8epv59or6g1b/v2, and is included for printing with this article as S1 Protocol. The detailed information on the materials that we used in this study is presented in S1 Table.

### Animals

RiboTag mice (stock 011029: B6N.129-*Rpl22*^*tm1*.*1Psam*^/J), Tie2-Cre mice (stock 008863: B6. Cg-Tg (Tek-cre) 1Ywa/J), and Ai9 mice (stock 007909: B6. Cg-*Gt (ROSA) 26Sortm9* ^*(CAG-tdTomato) Hze*^/J) were purchased from Jackson Laboratory. All mice used in this study were housed in a pathogen-free facility at the Korea Brain Research Institute Laboratory Animal Center. Eight- to twelve-week-old male *Tie2-Cre; Ai9; Rpl22*^*HA/HA*^ mice were used to collect brain endothelial cell-enriched mRNA. All animal experiments were performed according to approved Institutional Animal Care and Use Committee (IACUC-18-00009, 19–00002) protocols of the Korea Brain Research Institute.

### Immunoprecipitation

Brains from *Tie2-Cre; Rpl22*^*HA/HA*^ mice were lysed in a buffer consisting of 100 mM Tris-HCl (pH 7.5), 100 mM EDTA, 150 mM NaCl, and 1% Triton X-100 with freshly added Halt™ Protease and Phosphatase Inhibitor Cocktail (Thermo Fisher Scientific, 78444). Cell lysates were centrifuged at 12,000 × g for 10 min at 4°C, and the supernatants were then incubated with either anti-HA antibodies (1:200, Millipore, 05–904) or anti-Flag antibodies (1:200, Sigma, F1804) at 4°C overnight. The following day, the protein lysates were incubated with Protein A/G magnetic beads (Thermo Fisher Scientific, 88803) for 1 h at 4°C. Next, the beads were washed five times with lysis buffer, and the bound proteins were eluted with 2× SDS sample buffer by heating the beads at 95°C for 5 min. The samples were then analyzed by SDS–PAGE and Western blotting with anti-HA (1:200, Millipore, 05–904).

### Quantitative RT–PCR

The BEC specificity of the amplified cDNA was assessed by RT–PCR and quantitative RT–PCR (RT–qPCR) using LightCycler 480 SYBR Green I Master (Roche, 04707516001) and primers (brain endothelial cell markers: *Tie2*, *Mfsd2a*, *Claudin-5*, *Occludin*, *VCAM-1*, *P-gp*, neuronal cell marker: *Syt1*, astrocyte marker: *Gfap*, and pericyte marker: *Pdgfrβ*).

### Immunohistochemistry

To confirm Cre recombinase expression in *Tie2-Cre; Ai9; Rpl22*^*HA/HA*^ mice, brains were fixed in a 4% paraformaldehyde solution overnight. Mouse brain sections were then cut into 50 μm thick sections with a vibratome (Leica, VT200S). The mouse brain sections were permeabilized in PBST (PBS containing 0.2% Triton X-100) for 10 min, blocked with 2% BSA and 5% normal donkey serum in PBST for 60 min at room temperature, and then incubated in primary antibodies diluted with 2% BSA in PBST overnight at 4°C. The primary antibodies included anti-RFP (1:500, Thermo Fisher Scientific, MA5-15257), anti-CD31 (1:100, BD Biosciences, 553370), anti-GFAP (1:500, Agilent, Z0334), and anti-Pdgfrβ (1:500, Abcam, ab32570). After three cycles of washing with PBST (PBS containing 0.2% Tween 20) for 5 min, the sections were incubated for 1 h with Alexa Fluor 488-, or 594-conjugated-secondary antibodies (1:1000, Invitrogen). The sections were imaged with a fluorescence microscope (Nikon Eclipse Ti-U) or a Leica TCS SP8 Confocal Microscope (Leica, Germany).

## Results and discussion

**Fig 1:**
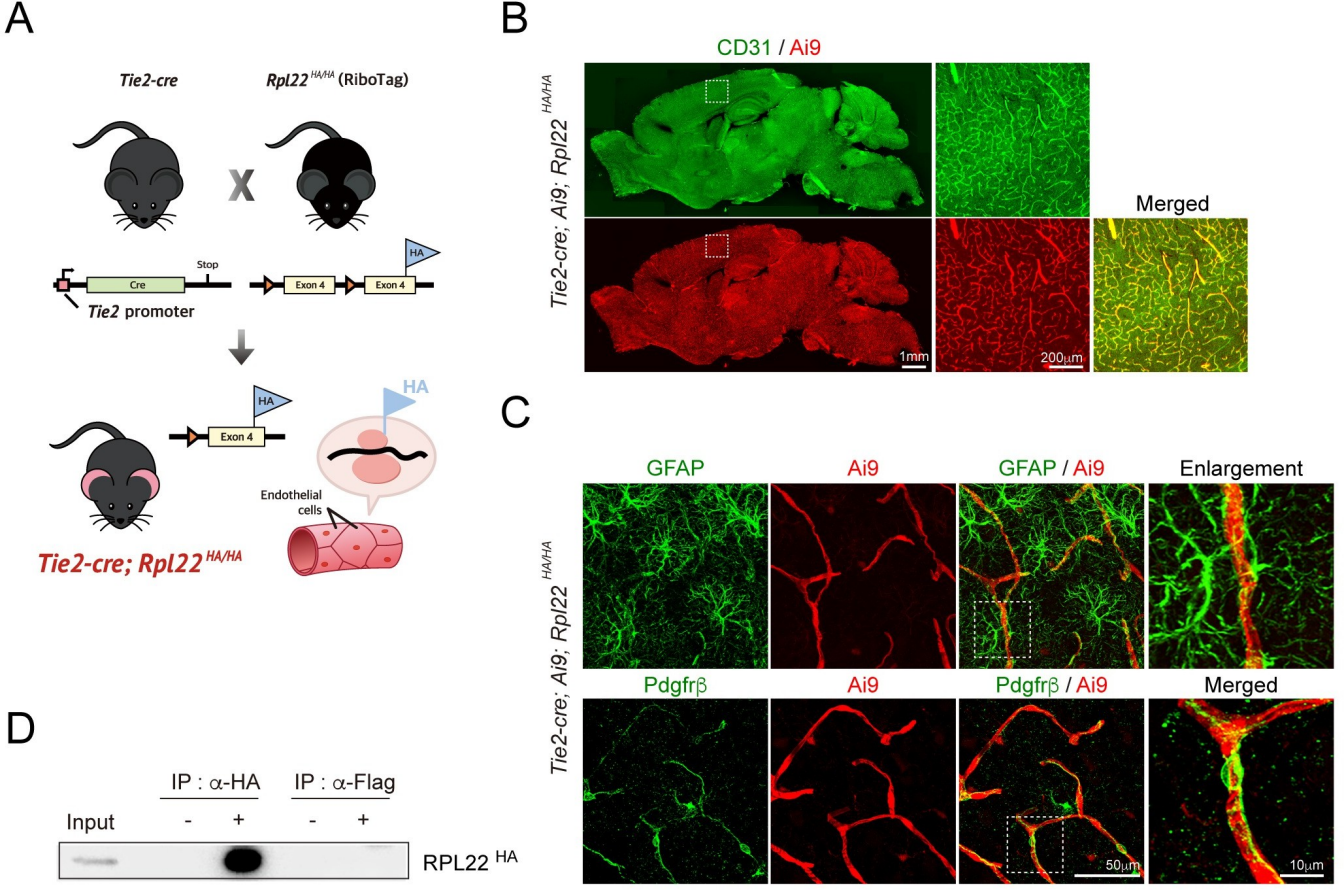
Validation of RiboTag mice for BEC-specific translatome analysis. **(A)** *Tie2-Cre; Rpl22*^*HA/HA*^ (*Ribo-Tag)* mice, which are designed for expressing HA-tagged ribosomal protein in BECs. **(B)** Immunohistochemistry for Ai9 (Cre reporter, Red) and CD31 (endothelial cell marker, Green) in adult *Tie2-Cre; Ai9; Rpl22*^*HA/HA*^ mice. The regions in the dotted boxes in the left images are shown on the right at higher resolution. Scale bars, 1 mm (200 μm in the insets). **(C)** Immunohistochemical staining for *Gfap* and Ai9 (left column) and for *Pdgfrb* and Ai9 (right column) in the cortex of adult *Tie2-Cre; Ai9; Rpl22*^*HA/HA*^ mice. *Gfap*+ astrocytes and *Pdgfr+* pericytes did not overlap with Cre recombinase, as represented by the Ai9 reporter gene. **(D)** Immunoprecipitation of the HA-tagged ribosomal protein RPL22HA, which is a component of actively translating polyribosomes. The expression of HA-tagged RPL22A was investigated by immunoprecipitation with mouse IgG (-) or anti-HA (+) or with anti-Flag (+) and subsequent Western blotting with anti-HA.

We aimed to develop an optimized RiboTag protocol for translatome data, which refers to mRNAs associated with actively translating ribosomes, in mouse cortex BECs. RiboTag mice, which were designed to express HA-tagged RPL22, a ribosomal protein dependent on Cre recombinase activity, were crossed with *Tie2-Cre* mice that express Cre recombinase under the *Tie2* promoter (Fig 1A). To test Cre activity and specificity, we crossed a Tie2-Cre mouse with the Ai9 reporter line, which is designed to express the tdTomato gene by eliminating the floxed STOP cassette under Cre activity. As shown in Fig 1B, tdTomato expression completely overlapped with CD31, which is a specific marker of endothelial cells. Furthermore, *Gfap*-positive astrocytes and *Pdgfrβ*-positive pericytes were not colocalized with Cre recombinase, as shown by the Ai9 reporter gene (Fig 1C). Taken together, these results suggest that Tie2 promoter-driven Cre recombinase is expressed specifically in most BECs. Next, we considered whether endothelial cell-specific expression of Cre recombinase could also lead to the expression of HA-tagged RPL22A. To test this hypothesis, we lysed the whole cortex of eight- to twelve-week-old male *Ti2-Cre; Ai9; Rpl22*^*HA/HA*^ mice and performed immunoprecipitation with anti-HA antibodies as described previously [10, 11]. As shown in Fig 1D, HA-tagged RPL22A proteins were successfully immunoprecipitated with anti-HA, not with anti-Flag or the IgG negative control.

**Fig 2:**
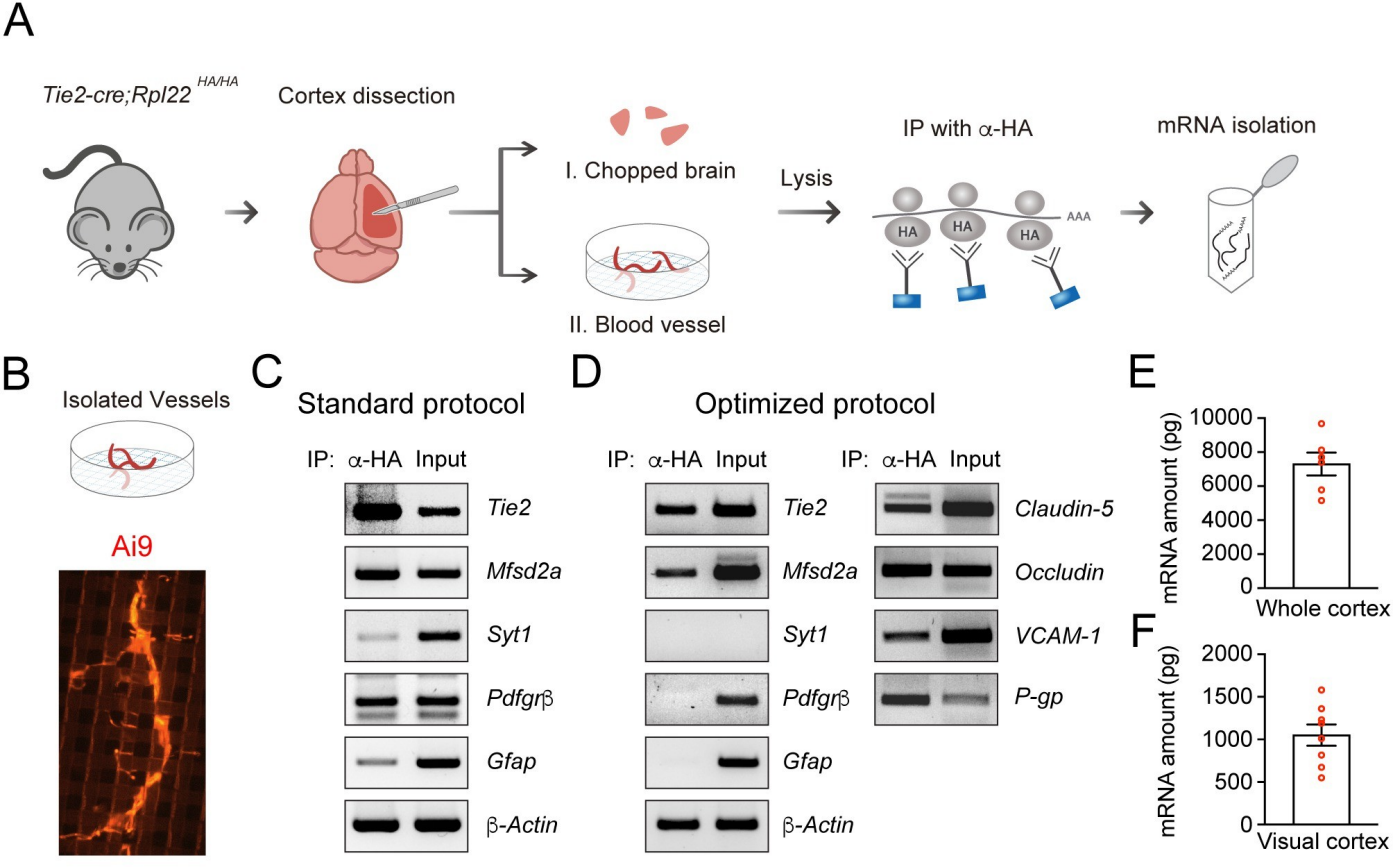
Comparative analysis of the conventional RiboTag method and our new RiboTag protocol. **(A)** Workflow diagram and validation of the RiboTag method. **(B)** Images of vessels isolated with a cell strainer (40 μm) before the immunoprecipitation step. **(C, D)** RT–PCR to verify the BEC specificity of mRNA pools isolated by the standard RiboTag protocol **(C)** and by our optimized RiboTag protocol **(D).** Brain endothelial cell markers: *Tie2*, *Mfsd2a*, *Claudin-5*, *Occludin*, *VCAM-1*, *P-gp*, neuronal cell marker: *Syt1*, astrocyte marker: *Gfap*, and pericyte marker: *Pdgfrβ*. The amount of mRNA isolated from the whole cortex (7.3 ± 0.668 ng) **(E)** and the visual cortex (1.05 ± 0.125 ng) **(F)**. Mean ± SEM; n = 6 for the whole cortex, n = 8 for the visual cortex.

To perform standard BEC translatome analysis, we carried out the RiboTag method with a standard protocol [10]. Briefly, we dissected the whole cortex from one *Ti2-Cre; Ai9; Rpl22*^*HA/HA*^ mouse and homogenized the brain tissues (Fig 2A). After mRNA isolation, we performed RT–PCR to verify BEC specificity (Fig 2C). Unfortunately, non-BEC markers, including *Syt1* for neuronal cells, *Pdgfrb* for pericytes, and *Gfap* for astrocytes, were also detected in the mRNA isolated with the original RiboTag method. To remedy this lack of specificity, we sought to establish a simplified and efficient protocol for a BEC-specific RiboTag method. We speculated that due to the cellular diversity and architectural complexity of the brain [12, 13], immunoprecipitation with the whole cortex increased the probability of obtaining mRNAs derived from non-BECs. Therefore, to obtain high-quality translating mRNA from BECs, two conditions must be satisfied: (1) elimination of brain tissues containing non-BEC cell types and (2) minimization of mRNA purification steps to preserve intact translating mRNA. Considering both requirements, we isolated blood vessels from the brain with a cell strainer (40 μm) (Fig 2A). After homogenization of the whole cortex from eight- to twelve-week-old male *Ti2-Cre; Ai9; Rpl22*^*HA/HA*^ mice, blood vessels were trapped with the cell strainer, which was confirmed by using a fluorescence microscope (Fig 2B). Following immunoprecipitation using the isolated vessels as input, the purity of the mRNA pools was validated by using RT–PCR (Fig 2D). Of interest, we were able to eliminate most mRNAs from neuronal cells through the blood vessel isolation procedure despite the presence of some traces from pericytes and astrocytes. However, most such non-BEC contamination was significantly reduced by the anti-HA immunoprecipitation step, and the average amount of BEC mRNA isolated from the whole cortex and visual cortex per mouse brain was 7.3 ± 0.668 ng (Fig 2E) and 1.05 ± 0.125 ng (Fig 2F), respectively. These results show that we developed a new optimized protocol using RiboTag mice to provide BEC-specific mRNA pools, despite the low mRNA yield.

**Fig 3:**
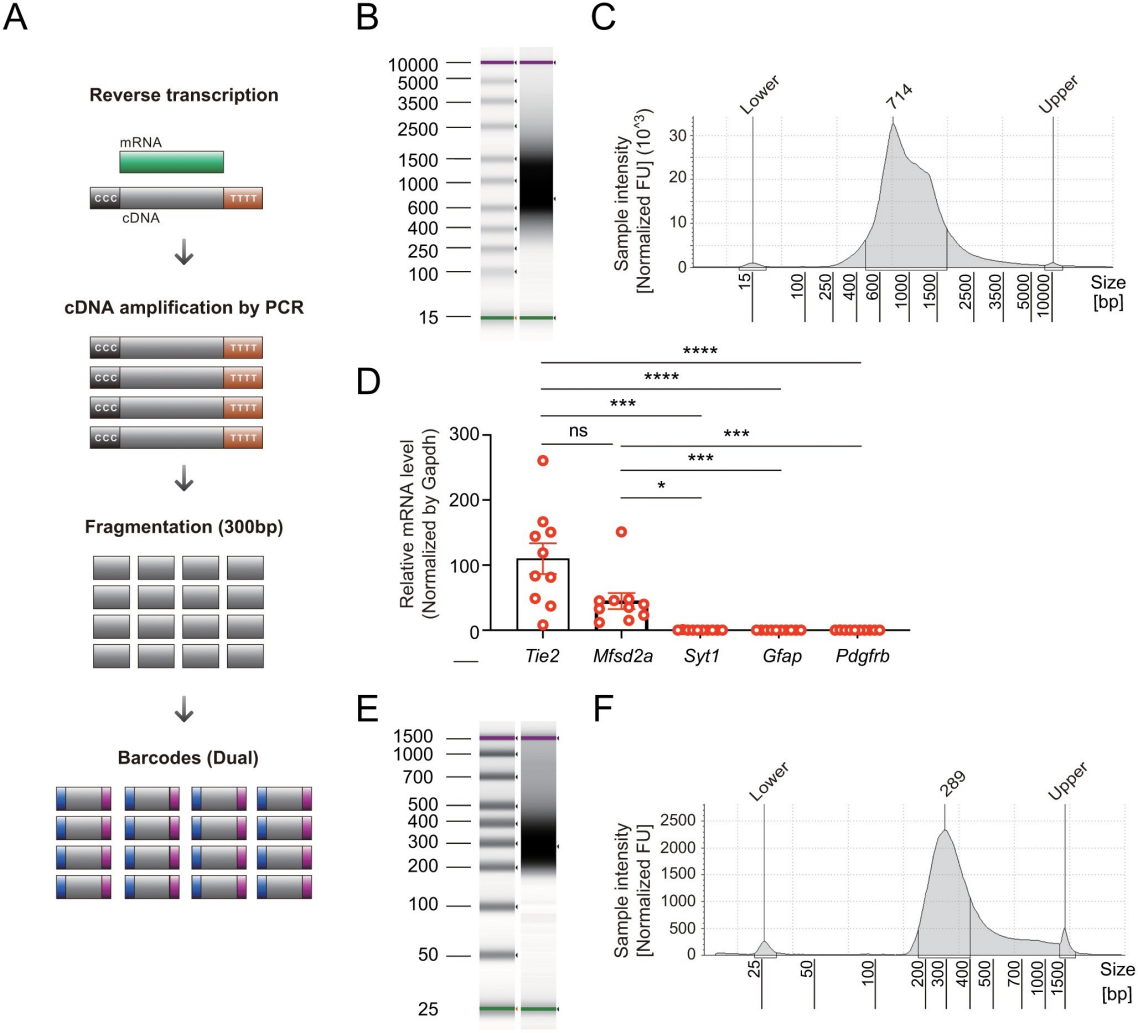
Generation of cDNA libraries for NGS from the low mRNA yield derived from BECs. **(A)** The process used to generate a cDNA library for RNA-seq from the small amount of pooled mRNA originating from BECs in the cortex of *Tie2-Cre; Ai9; Rpl22*^*HA/HA*^ mice. TapeStation gel image **(B)** and electropherogram **(C)** showing PCR amplification of the cDNA library from the cortex of *Tie2-Cre; Ai9; Rpl22*^*HA/HA*^ mice. **(D)** The specificity of the cDNA library from the cortex of *Tie2-Cre; Ai9; Rpl22*^*HA/HA*^ mice was verified by using RT–qPCR. Mean ± SEM; *P<0.05, ***P<0.001, ****P<0.0001 by the Kruskal–Wallis test, n = 10. After fragmentation into 300 bp pieces and labeling with dual barcode oligos, the quality of the cDNA was validated by using TapeStation gel images **(E)** and electropherograms **(F)**.

Although our optimized protocol obtains highly specific mRNA, there are potential limitations to applying this strategy for bulk gene expression analysis due to the low mRNA yield. Thus, we optimized a protocol for the generation of cDNA libraries by using the NEBNext Single Cell/Low Input RNA Library Prep Kit for Illumina (Fig 3A), which is applicable to NGS experiments to analyze the translatome in BECs. In testing the PCR amplification step for cDNA, we optimized the cycle number to 32 for the generation of a cDNA library from 1 ng of mRNA. Finally, we successfully generated a cDNA library (Fig 3B and 3C) and validated the BEC specificity of the amplified cDNA by using RT–qPCR (Fig 3D). Moreover, we labeled the cDNA library with dual barcode oligos after fragmentation into 300 bp pieces and successfully obtained cDNA libraries labeled with dual barcodes for NGS (Fig 3E and 3F).

## Supporting information

S1 Protocol (PDF)

S1 Table Key resources table. (PDF)
