# Supplementary material for: Optimized protocol for translatome analysis of mouse brain endothelial cells
Source: PLoS One. 2022 Sep 28;17(9):e0275036. doi: 10.1371/journal.pone.0275036 (PMC9518886; doi:10.1371/journal.pone.0275036)
Supplement: S1 Protocol — (PDF) [file pone.0275036.s001.pdf]

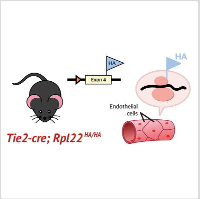

Version 2

Aug 24, 2022

Optimized protocol for translatome analysis of mouse brain endothelial cells V.2

Won-Jong OH[1], Namsuk Kim[1], Mi-Hee Jun[1], Jin-Young Jeong[1]

[1]Korea Brain Research Institute

1 Works for me Share

dx.doi.org/10.17504/protocols.io.8epv59or6g1b/v2

N Neurovascularlab KBRI

ABSTRACT

Brain endothelial cells (BECs) are important conduits that deliver oxygen and nutrients, protect parenchyma cells from toxins, and drain wastes to maintain brain homeostasis. Impairment of BECs has been implicated in diverse neurodegenerative diseases, including Alzheimer's disease and Parkinson's disease. Therefore, molecular analysis of BECs is important for understanding the molecular pathogenesis of these neurological diseases. Even though many transcriptome analyses for BECs have been developed, mRNA levels do not necessarily correlate with the levels of actively translated proteins. Translatome analysis using RiboTag mice, in which Rpl22, a ribosomal component, is tagged by the hemagglutinin epitope under Cre recombinase activation, could serve as an excellent tool that overcomes these caveats.However, implementation of this technique is limited by high noise-to-signal ratios as well as the low yield of mRNAs from BECs, which limits bulk gene expression analysis. In this study, we established a protocol to isolate highly pure mRNAs from BECs in the cortex of eight- to twelve-week-old male *Tie2-Cre; Rpl22*$^{HA/HA}$ mice by using a cell strainer to trap blood vessels prior to immunoprecipitation. According to the results of RT–PCR, the specificity of the mRNA pools isolated by our protocol was much higher than that of the pools isolated by the standard protocol. We were also able to generate a high-quality cDNA library for RNA-seq with the small amount of mRNA isolated with our protocol. Thus, this optimized method will be useful for future studies of BECs at the molecular level.

DOI

dx.doi.org/10.17504/protocols.io.8epv59or6g1b/v2

PROTOCOL CITATION

Won-Jong OH, Namsuk Kim, Mi-Hee Jun, Jin-Young Jeong 2022. Optimized protocol for translatome analysis of mouse brain endothelial cells. **protocols.io** https://protocols.io/view/optimized-protocol-for-translatome-analysis-of-mou-cfnytmfw
Version created by Neurovascularlab KBRI

Citation: Won-Jong OH, Namsuk Kim, Mi-Hee Jun, Jin-Young Jeong Optimized protocol for translatome analysis of mouse brain endothelial cells https://dx.doi.org/10.17504/protocols.io.8epv59or6g1b/v2

This is an open access protocol distributed under the terms of the **Creative Commons Attribution License** (https://creativecommons.org/licenses/by/4.0/), which permits unrestricted use, distribution, and reproduction in any medium,

KEYWORDS

Brain endothelial cells, RiboTag, Translatome, RNA sequencing

LICENSE

This is an open access protocol distributed under the terms of the Creative Commons Attribution License, which permits unrestricted use, distribution, and reproduction in any medium, provided the original author and source are credited

CREATED

Aug 23, 2022

LAST MODIFIED

Aug 24, 2022

PROTOCOL INTEGER ID

69048

GUIDELINES

1. An RNase-free environment is essential. Use barrier pipet tips to avoid RNase contamination. Wipe down the surface of an experimental table and all equipment including surgical tools, pipets, etc., with RNase Zap.

2. Homogenization buffer and high-salt buffer should be freshly prepared.

3. Washes should be done in cold conditions.

4. Tissue samples should be processed fresh directly to RNA yield.

5. The average amount of BEC mRNA => whole cortex (8-12 weeks): 7.3 ng, visual cortex (8-12 weeks): 1.05 ng, visual cortex (2 weeks): 0.25 ng per mouse.

MATERIALS TEXT

A	B	C
**REAGENT or RESOURCE**	**SOURCE**	**IDNETIFIER**
**Antibodies**		
Mouse anti-HA	Millipore	Cat# 05-904 RRID: AB_417380
**Chemicals, peptides, and kits**		
TRIzolTM Reagent	Thermo	Cat# 15596026

Citation: Won-Jong OH, Namsuk Kim, Mi-Hee Jun, Jin-Young Jeong Optimized protocol for translatome analysis of mouse brain endothelial cells https://dx.doi.org/10.17504/protocols.io.8epv59or6g1b/v2

This is an open access protocol distributed under the terms of the **Creative Commons Attribution License** (https://creativecommons.org/licenses/by/4.0/), which permits unrestricted use, distribution, and reproduction in any medium,

Cycloheximide	Sigma-Aldrich	Cat# 1810
Magnesium chloride	Sigma-Aldrich	Cat# M8266
Potassium chloride	Sigma-Aldrich	Cat# P9333
DNase1	Invitrogen	Cat# 18068015
Pierce™ Protein A/G Magnetic Beads	Thermo	Cat# 88803
Chloroform	Sigma	Cat# C2432
20X TE Buffer (pH 7.5)	Promega	Cat# A2651
Ethyl alcohol, Pure	Sigma	Cat# E7023
Glycogen, Molecular Biology Grade	Roche	Cat# 10901393001
RNasin® Ribonuclease Inhibitor	Promega	Cat# N2115
Pierce™ Protein A/G Magnetic Beads	Thermo Fisher Scientific	Cat# 88803
Halt™ Protease and Phosphatase Inhibitor Cocktail	Thermo Fisher Scientific	Cat# 78444
NEBNext® Single Cell/Low Input RNA Library Prep Kit for Illumina®	NEB	Cat# E6420L
NEBNext® Multiplex Oligos for Illumina®	NEB	Cat# E7600S
High Sensitivity D5000 Screen tape	Agilent	Cat# 5067-5592
High Sensitivity D1000 Screen tape	Agilent	Cat# 5067-5584
High Sensitivity RNA Screentape	Agilent	Cat# 5067-5579
High Sensitivity D5000 Screen tape Reagent	Agilent	Cat# 5067-5593
High Sensitivity D1000 Screen tape Reagent	Agilent	Cat# 5067-5585
High Sensitivity RNA Screen tape Reagent	Agilent	Cat# 5067-5580
High Sensitivity D5000 Screen tape ladder	Agilent	Cat# 5067-5594
High Sensitivity D1000 Screen tape ladder	Agilent	Cat# 5067-5587
High Sensitivity RNA Screen tape ladder	Agilent	Cat# 5067-5581
Glass homogenizer	WHEATON	Cat# 357542
Disposable scalpel	Bard-Parker	Cat# 371611
**Experimental models: Organisms/strains**		
Mouse: Tie2-Cre	The Jackson Laboratory	Stock# 008863
Mouse: Ai9	The Jackson Laboratory	Stock# 007909
Mouse:RiboTag mice (Rpl22HA/HA)	The Jackson Laboratory	Stock# 011029

Citation: Won-Jong OH, Namsuk Kim, Mi-Hee Jun, Jin-Young Jeong Optimized protocol for translatome analysis of mouse brain endothelial cells https://dx.doi.org/10.17504/protocols.io.8epv59or6g1b/v2

This is an open access protocol distributed under the terms of the **Creative Commons Attribution License** (https://creativecommons.org/licenses/by/4.0/), which permits unrestricted use, distribution, and reproduction in any medium,

SAFETY WARNINGS

TRIzol is a highly corrosive and toxic chemical that can cause burns on contact with the skin as well as systemic poisoning.

Chloroform can cause a person to become unconscious and even be fatal at high doses.

BEFORE STARTING

1. An RNase-*free* environment is essential. Use barrier pipet tips to avoid RNase contamination. Wipe down the surface of an experimental table and all equipment including surgical tools, pipets, etc., with RNase Zap.

2. Homogenization buffer and high-salt buffer should be freshly prepared.

Vessel isolation 2h

1 The whole mouse cortex of a *Tie2-Cre; Rpl22*$^{HA/HA}$ mouse is isolated in the chilled DMEM. Then, tissues are dissociated by using a glass homogenizer (WHEATON, 357542) in **10 mL** of chilled DMEM. 10m

2 **1000 x g, 4°C, 00:10:00** 10m

3 After discarding the supernatants, the pellets are resuspended in **15 mL** of 20 % BSA-DMEM to avoid myelin contamination. 5m

4 **2500 x g, 4°C, 00:15:00** 15m

5 **This process is repeated three times.** 40m

Citation: Won-Jong OH, Namsuk Kim, Mi-Hee Jun, Jin-Young Jeong Optimized protocol for translatome analysis of mouse brain endothelial cells https://dx.doi.org/10.17504/protocols.io.8epv59or6g1b/v2

This is an open access protocol distributed under the terms of the **Creative Commons Attribution License** (https://creativecommons.org/licenses/by/4.0/), which permits unrestricted use, distribution, and reproduction in any medium,

6 After discarding the supernatants, the pellets are resuspended in 5 mL of chilled PBS. 5m

7 PBS containing blood vessels is passed through a 40-micrometer cell strainer. 5m

Immunoprecipitation 1d 1h

8 The strainer mesh containing vessels is then cut with a disposable scalpel (Bard-Parker, 371611) and transferred into a microcentrifuge tube for lysis in 600 µL of homogenization buffer containing [M]1 % (v/v) NP-40, [M]100 millimolar (mM) KCl, [M]50 millimolar (mM) Tris (pH 7.4), [M]12 millimolar (mM) $MgCl_2$, cycloheximide ([M]100 mg/mL), heparin ([M]1 mg/mL), Halt Protease and Phosphatase Inhibitor Cocktail (Thermo Fisher Scientific, 78444), RNA inhibitor (5 units/ml, Promega, N2615), and [M]1 millimolar (mM) DTT). 5m

9 The lysates are incubated On ice for 00:05:00 . 5m

10 12000 x g, 4°C, 00:10:00 10m

11 After being transferred to a new 1.5 ml microcentrifuge tube, the supernatants are incubated with a mouse monoclonal antibody against the HA epitope tag (1:200, Millipore, 05-904) for 04:00:00 at 4 °C with rotation by using a multimixer (NanoEnTek, 4519). 4h

.

12 Protein A/G magnetic beads (Thermo Fisher Scientific, 88803) equilibrated in homogenization buffer for 30 min are added to the antibody-lysate solution and incubated Overnight at 4 °C with gentle rotation. 16h

13 The next day, after a brief spin-dwon, the magnetic beads are washed five times with 1 mL high salt buffer (1% NP-40, [M]300 millimolar (mM) KCL, [M]50 millimolar (mM) Tris (pH 7.4), [M]12 millimolar (mM) $MgCl_2$, cycloheximide ([M]100 mg/mL), and 30m

Citation: Won-Jong OH, Namsuk Kim, Mi-Hee Jun, Jin-Young Jeong Optimized protocol for translatome analysis of mouse brain endothelial cells https://dx.doi.org/10.17504/protocols.io.8epv59or6g1b/v2

This is an open access protocol distributed under the terms of the **Creative Commons Attribution License** (https://creativecommons.org/licenses/by/4.0/), which permits unrestricted use, distribution, and reproduction in any medium,

[M]0.5 millimolar (mM) DTT).

mRNA isolation 5h 55m

14 After the last wash, all supernatants are removed and 1 mL of TRIzol reagent (Invitrogen, 15596026) is added to the bead-antibody-tissue homogenate, followed by 200 µL of chloroform (Sigma-Aldrich, C2432). 10m

> TRIzol is a highly corrosive and toxic chemical that can cause burns on contact with the skin as well as systemic poisoning.
>
> Chloroform can cause a person to become unconscious and even be fatal at high doses.

15 12000 x g, 4°C, 00:10:00 10m

16 The upper aqueous layer (approximately 600 µL) is transferred into a new 15 ml conical tube, and 60 µL of [M]4 Molarity (M) LiCl, 120 µL of 20 X TE ([M]0.2 Molarity (M) Tris-HCl, [M]20 millimolar (mM) EDTA, pH 7.5 , Promega, A2651), 1.8 mL of 100% ethyl alcohol (Sigma, E7023), and 3 mL of glycogen (Roche, 10901393001) are added for RNA precipitation. 15m

17 The mRNA mixture is incubated Overnight at -20 °C 16h

18 The following day, samples are centrifuged 12000 x g, 4°C, 00:10:00 . After the supernatants are discarded, 1 mL of 75% ethyl alcohol is added to the pellets for washing. 10m

19 After centrifugation 7500 x g, 4°C, 00:05:00 and subsequent supernatant removal, the 10m

Citation: Won-Jong OH, Namsuk Kim, Mi-Hee Jun, Jin-Young Jeong Optimized protocol for translatome analysis of mouse brain endothelial cells https://dx.doi.org/10.17504/protocols.io.8epv59or6g1b/v2

This is an open access protocol distributed under the terms of the **Creative Commons Attribution License** (https://creativecommons.org/licenses/by/4.0/), which permits unrestricted use, distribution, and reproduction in any medium,

samples are air-dried for 00:05:00 at Room temperature . Do not overdry the beads.

20 The dried pellets are then resuspended in 16 µL of RNase-free water. 5m

21 2 µL of DNase I and 2 µL of 10X DNase I Reaction Buffer (Invitrogen, 18068-015) are added to the reaction mixture, which is then incubated for 00:15:00 at Room temperature . 15m

22 DNaseI is inactivated by adding [M]25 millimolar (mM) of EDTA and heating at 65 °C for 00:10:00 . 10m

23 For RNA precipitation, 2.2 µL of [M]4 Molarity (M) of LiCl, 4.8 µL of 20 X TE ([M]0.2 Molarity (M) Tris-HCl, [M]20 millimolar (mM) EDTA, pH 7.5 (Promega, A2651), 66 µL of 100% ethyl alcohol (Sigma-Aldrich, E7023), and 1 µL of glycogen (Roche, 10901393001) are added to the RNA mixture, followed by Overnight incubation at -20 °C . 16h

24 The next day, the RNA mixture is centrifuged 12000 x g, 4°C, 00:10:00 10m

25 After removing the supernatants, 1 mL of 75% ethyl alcohol is added to the pellets for washing. 5m

26 After centrifugation 7500 x g, 4°C, 00:05:00 , the supernatants are discarded. 5m

27 The pellets are then air-dried and finally resuspended in 10 µL of RNase-free water. 5m

Generation of cDNA library 2h 25m

Citation: Won-Jong OH, Namsuk Kim, Mi-Hee Jun, Jin-Young Jeong Optimized protocol for translatome analysis of mouse brain endothelial cells https://dx.doi.org/10.17504/protocols.io.8epv59or6g1b/v2

This is an open access protocol distributed under the terms of the **Creative Commons Attribution License** (https://creativecommons.org/licenses/by/4.0/), which permits unrestricted use, distribution, and reproduction in any medium,

28 The amount of isolated mRNA is measured by using High Sensitivity RNA ScreenTape (Agilent, 5067-5579), High Sensitivity RNA ScreenTape Reagent(Agilent, 5067-5580), and a High Sensitivity RNA ScreenTape ladder(Agilent, 5067-5581) from the Agilent 4200 TapeStation System according to the manufacturer's instructions.

29 One nanogram of mRNA obtained from RiboTag immunoprecipitation is reverse-transcribed into cDNA using the NEBNext Single Cell/Low Input RNA Library Prep Kit for Illumina (NEB, E6420L) according to the manufacturer’s protocol.

30 One nanogram of mRNA is added to the mixture containing **1 µL** of NEBNext Single Cell RT (Reverse Transcription) Primer Mix. The **9 µL** of the final volume is achieved by adding nuclease-free water.

31 The mixture is incubated at **70 °C** for **00:05:00** with the heated lid set to **105 °C** for annealing and then held at **4 °C** . 5m

32 The RT mixture is prepared in a separate tube as follows **On ice** ; **5 µL** of NEBNext Single Cell RT buffer, **1 µL** of NEBNext Template Switching Oligo, **2 µL** of NEBNext Single Cell RT Enzyme Mix, **3 µL** of nuclease-free water. It is important to vortex the NEBNext Single Cell RT buffer prior to use for optimal performance.

33 The RT mixture (**11 µL**) is combined with the annealed sample (**9 µL**). Mix well by pipetting up and down at least 10 times.

34 The reaction is incubated in a thermocycler with the following steps: the heated lid is set to **105 °C** , followed by **01:30:00** at **42 °C** and **00:10:00** at **70 °C** , and then held at **4 °C** 1h 40m

35 The cDNA amplification mix is prepared as follows: **50 µL** of NEBNext Single Cell cDNA PCR Master Mix, **2 µL** of NEBNext Single Cell cDNA PCR Primer, and **28 µL** of nuclease-free water.

36 **80 µL** of cDNA amplification mix are added to **20 µL** of the sample with pipetting.

Citation: Won-Jong OH, Namsuk Kim, Mi-Hee Jun, Jin-Young Jeong Optimized protocol for translatome analysis of mouse brain endothelial cells https://dx.doi.org/10.17504/protocols.io.8epv59or6g1b/v2

This is an open access protocol distributed under the terms of the **Creative Commons Attribution License** (https://creativecommons.org/licenses/by/4.0/), which permits unrestricted use, distribution, and reproduction in any medium,

37 The reaction is performed in a thermocycler with the following PCR cycling conditions.

Cycle step	Temperature	Time	Cycles
Initial Denaturation	98 °C	45 sec	1
Denaturation	98 °C	10 sec	32
Annealing	62 °C	15 sec	
Extension	72 °C	3 min	
Final Extension	72 °C	5 min	1
Hold	4 °C		

38 For the next step, the NEBNext Bead Reconstitution Buffer and the SPRI (Solid Phase Reversible Immobilization) beads should be warmed to **Room temperature** for at least **00:30:00** before use. 30m

39 **60 µL** SPRI beads are added to the PCR. (mix well by pipetting up and down at least 10 times).

40 The samples are incubated for at least **00:05:00** at **Room temperature** . 5m

41 The samples are placed on the magnetic stand (Promega, Z5342) to separate the beads from the supernatant.

42 After **00:05:00** , the supernatant is removed. then, **200 µL** of 80% freshly prepared ethanol is added for washing. The samples are Incubated at **Room temperature** for **00:00:30** , and then the supernatant is carefully removed and discarded. 5m 30s

43 . This process is repeated twice. The samples are air-dried for **00:05:00** at **Room temperature** . Do not overdry the beads. 5m

Citation: Won-Jong OH, Namsuk Kim, Mi-Hee Jun, Jin-Young Jeong Optimized protocol for translatome analysis of mouse brain endothelial cells https://dx.doi.org/10.17504/protocols.io.8epv59or6g1b/v2

This is an open access protocol distributed under the terms of the **Creative Commons Attribution License** (https://creativecommons.org/licenses/by/4.0/), which permits unrestricted use, distribution, and reproduction in any medium,

44 50 µL of 0.1X TE (diluted from 1X TE buffer) is added to the samples to elute the cDNA from the beads. 2m

The samples are mixed well and incubateed for at least 00:02:00 at Room temperature .

45 Next, 45 µL of NEBNext Bead Reconstitution Buffer is added to the cDNA-Bead mixture. 5m

Mix well by pipetting up and down at least 10 times and incubate for at least 00:05:00 at Room temperature .

46 The samples are placed on a magnetic stand to separate the beads.

47 After 00:05:00 , the supernatant is carefully removed. 5m

48 Then, 200 µL of 80% freshly prepared ethanol is added to the tube to wash the beads. 30s

After 00:00:30 of incubation at Room temperature , . This process is repeated twice.

49 The beads containing cDNA are air-dried for 00:05:00 at Room temperature . Do not overdry the beads. 5m

50 cDNA is eluted from the beads by adding 33 µL of 1X TE. Mix well by pipetting up and down at least 10 times. The sample is incubated for at least 00:02:00 at Room temperature . 2m

51 The sample is placed on the magnetic stand. After 00:05:00 of incubation at Room temperature , 30 µL of the solution is transferred to a new tube. 5m

52 The cDNA quality and quantity can be assessed by using High Sensitivity D5000 ScreenTape (Agilent, 5067-5592), High Sensitivity D5000 ScreenTape Reagent (Agilent, 5067-5593), and a

Citation: Won-Jong OH, Namsuk Kim, Mi-Hee Jun, Jin-Young Jeong Optimized protocol for translatome analysis of mouse brain endothelial cells https://dx.doi.org/10.17504/protocols.io.8epv59or6g1b/v2

This is an open access protocol distributed under the terms of the **Creative Commons Attribution License** (https://creativecommons.org/licenses/by/4.0/), which permits unrestricted use, distribution, and reproduction in any medium,

High Sensitivity D5000 ScreenTape ladder (Agilent, 5067-5594) in the Agilent 4200 TapeStation System.

53 40 ng of cDNA is used for Illumina NGS (Next Generation Sequencing) library preparation.

54 40 ng of cDNA in 1X TE is mixed with 7 µL of NEBNext Ultra II FS Reaction Buffer and 2 µL of NEBNext Ultra II FS Enzyme Mix in a PCR tube. The final volume of the mixture is brought to 35 µL , and the sample is vortexed for 00:00:05 . 5s

55 In a thermocycler, with the heated lid set to 75 °C , the following program is performed: 00:25:00 at 37 °C and 00:30:00 at 65 °C . 55m

56 While the PCR is running, prepare the solution for the next step. NEBNext Adaptor for Illumina is diluted by 25-fold in the NEBNext Adaptor Dilution Buffer.

57 The following components should be added directly to the above sample (35 µL). The adaptor should be added separately to each sample (DO NOT premix with ligation master mix and enhancer).

Component	Volume
FS Reaction Mixture	35 µl
NEBNExt Ultra II Ligation Master Mix	30 µl
NEBNext Ligation Enhancer	1 µl
NEBNext Adaptor for Illumina (dilluted 1:25)	2.5 µl

58 The samples are mixed well by using pipetting the entire volume up and down at least 10 times. The ligation mixture is incubated at 20 °C for 00:15:00 in a thermocycler without the heated lid. 15m

59 3 µL of USER Enzyme, a mixture of uracil DNA glycosylase (UDG) and the DNA glycosylase-lyase endonuclease VIII, is added to the ligation mixture and mixed well. Incubate 15m

Citation: Won-Jong OH, Namsuk Kim, Mi-Hee Jun, Jin-Young Jeong Optimized protocol for translatome analysis of mouse brain endothelial cells https://dx.doi.org/10.17504/protocols.io.8epv59or6g1b/v2

This is an open access protocol distributed under the terms of the **Creative Commons Attribution License** (https://creativecommons.org/licenses/by/4.0/), which permits unrestricted use, distribution, and reproduction in any medium,

at 37 °C for 00:15:00 .

60 For the next step, the NEBNext Bead Reconstitution Buffer and the SPRI beads should be warm to Room temperature for at least 00:30:00 before use. 30m

61 57 µL of SPRI beads are added to the PCR reaction. The sample is incubated for at least 00:05:00 at Room temperature . 5m

62 The sample is placed on a magnetic stand.

63 After 00:05:00 incubation, the supernatant is removed. Then, 200 µL of 80% freshly prepared ethanol is added to the tube. After incubation at Room temperature for 00:00:30 , the supernatant is removed. . This process is repeated twice. 5m 30s

64 The beads containing cDNA are air-dried for 00:05:00 at Room temperature . Do not overdry the beads. 5m

65 17 µL of 0.1X TE is added to resuspend the beads. The cDNA-bead mixture is incubated for at least 00:02:00 at Room temperature . 2m

66 The sample is placed on a magnetic stand. After 00:05:00 , 15 µL of the cleared solution is transferred to a new PCR tube. 5m

67 The following components are combined into a new PCR tube.

Component	Volume
Adaptor Ligated DNA Fragments	15 µl
NEBNext Ultra II Q5 Master Mix	25 µl
Index Primer / i7	5 µl
Index Primer / i5	5 µl

Citation: Won-Jong OH, Namsuk Kim, Mi-Hee Jun, Jin-Young Jeong Optimized protocol for translatome analysis of mouse brain endothelial cells https://dx.doi.org/10.17504/protocols.io.8epv59or6g1b/v2

This is an open access protocol distributed under the terms of the **Creative Commons Attribution License** (https://creativecommons.org/licenses/by/4.0/), which permits unrestricted use, distribution, and reproduction in any medium,

68 Labelling with dual barcodes is performed by using the following PCR cycling conditions.

Cycle step	Temperature	Time	Cycles
Initial Denaturation	98 °C	30 sec	1
Denaturation	98 °C	10 sec	8
Annealing	65°C	75 sec	
Final Extension	65 °C	5 min	
Hold	4 °C		

69 For the next step, the NEBNext Bead Reconstitution Buffer and the SPRI beads should be warmed to **Room temperature** for at least **00:30:00** before use. 30m

70 The PCR mixture is resuspended in **45 µL** of SPRI beads. The sample is incubated for at least **00:05:00** at **Room temperature** . 5m

71 The cDNA-bead mixture is placed on a magnetic stand to separate the beads from the supernatant.

72 After **00:05:00** , the supernatant is removed and discarded. 5m

73 **200 µL** of 80% freshly prepared ethanol are added to the tube in the magnetic stand. . This process is repeated twice.

74 The beads containing cDNA are air-dried on a magnetic stand for **00:05:00** at **Room temperature** . 5m

75 The cDNA library is eluted by adding **33 µL** of 0.1X TE. Mix well by pipetting up and down 10 times.

Citation: Won-Jong OH, Namsuk Kim, Mi-Hee Jun, Jin-Young Jeong Optimized protocol for translatome analysis of mouse brain endothelial cells https://dx.doi.org/10.17504/protocols.io.8epv59or6g1b/v2

This is an open access protocol distributed under the terms of the **Creative Commons Attribution License** (https://creativecommons.org/licenses/by/4.0/), which permits unrestricted use, distribution, and reproduction in any medium,

76 The sample is placed on a magnetic stand. After **00:05:00** , **30 µL** of the sample containing the cDNA library is transferred to a new tube. Libraries can be stored at **-20 °C** . 5m

77 Before NGS, the quality of the final cDNA libraries is checked by using High Sensitivity D1000 ScreenTape (Agilent, 5067-5584), High Sensitivity D1000 ScreenTape Reagent(Agilent, 5067-5585), and a High Sensitivity D1000 ScreenTape ladder (Agilent, 5067-5587) in the Agilent 4200 TapeStation System.

Citation: Won-Jong OH, Namsuk Kim, Mi-Hee Jun, Jin-Young Jeong Optimized protocol for translatome analysis of mouse brain endothelial cells https://dx.doi.org/10.17504/protocols.io.8epv59or6g1b/v2

This is an open access protocol distributed under the terms of the **Creative Commons Attribution License** (https://creativecommons.org/licenses/by/4.0/), which permits unrestricted use, distribution, and reproduction in any medium,
