# Supplementary material for: Optimized protocol for translatome analysis of mouse brain endothelial cells
Source: PLoS One. 2022 Sep 28;17(9):e0275036. doi: 10.1371/journal.pone.0275036 (PMC9518886; doi:10.1371/journal.pone.0275036)
Supplement: S1 Table — (PDF) [file pone.0275036.s002.pdf]

S2. Key resources table

REAGENT or RESOURCE	SOURCE	IDNETIFIER
**Antibodies**		
Rat anti-CD31	BD Biosciences	Cat# 553370<br>RRID: AB_394816
Mouse anti-RFP	Thermo Fisher Scientific	Cat# MA5-15257<br>RRID: AB_10999796
Rabbit anti-Pdgfrβ	Abcam	Cat# ab32570<br>RRID: AB_777165
Mouse anti-Gfap	Agilent	Cat# Z0334<br>RRID: AB_10013382
Mouse anti-HA	Millipore	Cat# 05-904<br>RRID: AB_417380
Mouse anti-Flag	Sigma	Cat# F1804<br>RRID: AB_ 262044
Donkey anti-rabbit IgG, Alexa Fluor 647	Thermo Fisher Scientific	Cat# A-31573<br>RRID:AB_ 2536183
Donkey anti-rat IgG, Alexa Fluor 488	Thermo Fisher Scientific	Cat# A-21208<br>RRID:AB_ 141709
Donkey anti-mouse IgG, Alexa Fluor 568	Thermo Fisher Scientific	Cat# A-10037<br>RRID:AB_2757558
Chemicals, peptides, and kits		
TRIzol^{TM} Reagent	Thermo Fisher Scientific	Cat# 15596026
Cycloheximide	Sigma-Aldrich	Cat# 1810
Magnesium chloride	Sigma-Aldrich	Cat# M8266
Potassium chloride	Sigma-Aldrich	Cat# P9333
DNase1	Invitrogen	Cat# 18068015
Paraformaldehyde	Electron Microscopy	Cat#19202

	Sciences	
Pierce™ Protein A/G Magnetic Beads	Thermo Fisher Scientific	Cat# 88803
Chloroform	Sigma	Cat# C2432
20X TE Buffer (pH 7.5)	Promega	Cat# A2651
Ethyl alcohol, Pure	Sigma	Cat# E7023
Glycogen, Molecular Biology Grade	Roche	Cat# 10901393001
LightCycler 480 SYBR Green I Master	Roche	Cat# 04 887 352 001
RNasin® Ribonuclease Inhibitor	Promega	Cat# N2115
Pierce™ Protein A/G Magnetic Beads	Thermo Fisher Scientific	Cat# 88803
Halt™ Protease and Phosphatase Inhibitor Cocktail	Thermo Fisher Scientific	Cat# 78444
NEBNext® Single Cell/Low Input RNA Library Prep Kit for Illumina®	NEB	Cat# E6420L
NEBNext® Multiplex Oligos for Illumina®	NEB	Cat# E7600S
High Sensitivity D5000 Screen tape	Agilent	Cat# 5067-5592
High Sensitivity D1000 Screen tape	Agilent	Cat# 5067-5584
High Sensitivity RNA Screentape	Agilent	Cat# 5067-5579
High Sensitivity D5000 Screen tape Reagent	Agilent	Cat# 5067-5593
High Sensitivity D1000 Screen tape Reagent	Agilent	Cat# 5067-5585
High Sensitivity RNA Screen tape Reagent	Agilent	Cat# 5067-5580
High Sensitivity D5000 Screen tape ladder	Agilent	Cat# 5067-5594
High Sensitivity D1000 Screen tape ladder	Agilent	Cat# 5067-5587
High Sensitivity RNA Screen tape ladder	Agilent	Cat# 5067-5581
ProLong™ Diamond Antifade Mountant with DAPI	Thermo Fisher Scientific	Cat# P36962
Glass homogenizer	WHEATON	Cat# 357542
Disposable scalpel	Bard-Parker	Cat# 371611

Experimental models: Organisms/strains		
Mouse: *Tie2-Cre*	The Jackson Laboratory	Stock# 008863
Mouse: *Ai9*	The Jackson Laboratory	Stock# 007909
Mouse: RiboTag mice ($Rpl22^{HA/HA}$)	The Jackson Laboratory	Stock# 011029
Oligonucleotides		
CCCTCCTCAACCAGAAAACA	N/A	Tie2 Forward
GCCCTTGAGCTGGTACTGAG	N/A	Tie2 Reverse
CGATGACTTCCACCTGAAACACC	N/A	Mfsd2a Forward
AACCTGAGCTGCTGGCTTCTTCT	N/A	Mfsd2a Reverse
CCAGAGCAGAGGCACCAG A	N/A	Claudin-5 Forward
AGACACAGCACCAGACCCAGA	N/A	Claudin-5 Reverse
AGATTCCTCTGACCTTGAGTGTGG	N/A	Occludin Forward
TCCTGCTTTCCCCTTCGTG	N/A	Occludin Reverse
GGAGAGACAAAGCAGAAGTGGA	N/A	VCAM-1 Forward
CACACGTCAGAACAACCGAATC	N/A	VCAM-1 Reverse
TCATTGCGATAGCTGGAG	N/A	P-gp Forward
CAAACTTCTGCTCCCGAGTC	N/A	P-gp Reverse
CTTTGCCATCTTCGCCTTTG	N/A	Syt1 Forward
TTTAACGCAGGAGGGTGCAT	N/A	Syt1 Reverse
AAGCTCCAAGATGAAACCAACCTGA	N/A	Gfap Forward
GCAAACTTAGACCGATACCACTC	N/A	Gfap Reverse
CACCTTCTCCAGTGTGCTGA	N/A	Pdgfrβ Forward
GGAGTCCATAGGGAGGAAGC	N/A	Pdgfrβ Reverse
TGACGTGCCGCCTGGAGAAAC	N/A	Gapdh Forward
CCGGCATCGAAGGTGGAAGAG	N/A	Gapdh Reverse

Software and algorithms		
Prism 9	GraphPad	https://www.graphpad.com/scientific-software/prism/
LightCycler®480 (v1.5.1)	Roche	https://lifescience.roche.com/
Leica Application Suite X	Leica	https://www.leica-microsystems.com/
NIS-Elements (v4.50.00)	Nickon	https://www.microscope.healthcare.nikon.com/
